# Intersecting Epidemics: The Predictors of Continued Utilization of HIV Care and Treatment Services During the COVID-19 Outbreak Among People Living with HIV in ZIMBABWE

**DOI:** 10.1007/s10461-023-04194-x

**Published:** 2023-10-20

**Authors:** Talent Tapera, Clifford Odimegwu, Tatenda Makoni, Waraidzo Mukuwapasi, Vivian Chitiyo, Gilton Kadziyanike, Nicola Willis, Abigail Mutsinze, Mather Mawodzeke, Pugie Chimberengwa, Million Phiri

**Affiliations:** 1 Demography and Population Studies Programme, Schools of Public Health and Social Sciences, University of the Witwatersrand, Johannesburg, South Africa; 2 Zimbabwe National Network of People Living With HIV (ZNNP+), Harare, Zimbabwe; 3 Zvandiri, Harare, Zimbabwe; 4 Organisation for Public Health Interventions and Development (OPHID), Harare, Zimbabwe; 5 Department of Population Studies, Schools of Humanities and Social Sciences, University of Zambia, Lusaka, Zambia

**Keywords:** HIV, COVID-19, Public health, Zimbabwe

## Abstract

Globally, there have been considerable achievements towards HIV care and treatment. AIDS-related deaths have been reduced by 60% since the peak in 2004. Potentially, the fight against the HIV epidemic was made more difficult with the outbreak of COVID-19. Thus, this study examined the implications of COVID-19 in the utilization of HIV care and treatment services among people living with HIV on antiretroviral therapy (ART) in Zimbabwe. The study aimed to identify the critical factors defining the utilization of HIV services at the advent of COVID-19 using the fifth revision of the Anderson Behavioral Model of Healthcare Utilization. The study utilized a concurrent triangulation design of which only one data collection phase was used. The quantitative data was collected from 2,157 people living with HIV on antiretroviral viral therapy through a structured interviewer-administered questionnaire. On the other hand, qualitative data was collected through in-depth interviews. Regarding accessing ART refills, the study findings revealed that adolescents aged 15–19 (aOR = 2.16; 95% CI: 1.18–3.96) had higher odds of utilizing ART refills compared to their counterparts who were aged 20–24. Living in a rural area was associated with higher odds of utilizing the ART refill service (aOR = 2.20; 95% CI: 1.49–3.24). Regarding accessing viral load monitoring adults aged 25–39 (aOR = 0.41; 95% CI: 0.26–0.66) were less likely to utilize viral load monitoring compared to young people aged 20–24. Being vaccinated for COVID-19 was significantly associated with higher odds of utilizing the viral load monitoring service (aOR = 1.97; 95% CI: 1.36–2.87) than those not yet vaccinated. Living in a rural area was associated with higher odds of utilizing viral load monitoring (aOR = 1.50; 95% CI: 1.09–2.08). Regarding tuberculosis preventative therapy, adults aged 25–39 (aOR = 0.30; 95% CI: 0.20–0.47) were less likely to utilize tuberculosis preventative therapy compared to young people aged 20–24. Being vaccinated for COVID-19 was significantly associated with higher odds of utilizing tuberculosis preventative therapy (aOR = 1.59; 95% CI: 1.12–2.25) than those not yet vaccinated. Living in a rural area was associated with higher odds of utilizing tuberculosis preventive therapy (aOR = 1.58; 95% CI: 1.19–2.08). Regarding tuberculosis screening being vaccinated for COVID-19 was significantly associated with higher odds of utilizing tuberculosis screening services (aOR = 1.89; 95% CI: 1.41–2.54) than those not yet vaccinated. Although the severity of the COVID-19 pandemic has dwindled, COVID-19 appears to come and go in waves, and a few countries are still recording relatively high cases. It is therefore likely that the factors associated with utilization of HIV services identified by the study such as age, residence, type of health facility, vaccination of COVID-19 and fear of contracting COVID-19, among others, need to be included when planning to improve access to health utilization.

## Introduction

The COVID-19 pandemic has had adverse effects on the utilization of the broader sexual and reproductive health (SRH) services. The supply chain for key commodities was disrupted by the delays in the manufacture of key pharmaceutical components of contraceptive methods and by delays in the transportation of contraceptive commodities [1]. This may also have impacted HIV testing, prevention, and treatment commodities. More so, the staff involved in the provision of SRH services had to fulfil other needs which could have included COVID-19 treatments and vaccinations [2]. Health facilities closed due to staff COVID-19 isolations, while people were also reluctant to go to health facilities for SRH services due to COVID-19 [2]. In addition, many governments restricted people’s movements due to lockdowns meant to thwart the spread of the COVID-19 virus [3]. Healthcare providers were forced to suspend some SRH services that are not classified as essential, such as abortion care, thus denying people this time-sensitive and potentially life-saving service [2].

Regarding the fight against the HIV epidemic, antiretroviral therapy (ART) transformed HIV from being a terminal illness to being a chronic disease [4]. As ART is a lifelong treatment, there is a need to ensure that people living with HIV are retained on treatment accessing the expected services to prevent and treat opportunistic infections [5]. Expected HIV care and treatment services include routinely receiving the ART drug refill, viral load monitoring, tuberculosis (TB) preventative therapy and TB screening and treatment, among others, for eligible clients. Tuberculosis (TB) screening is also a regular standard of care in HIV care and treatment for people living with HIV as they are at heightened risk of TB infection [6]. More so, TB preventative therapy works with ART to reduce TB morbidity and mortality [6]. The maximum utilization of HIV care and treatment services is critical in ensuring that people living with HIV maintain healthy lives, in reducing HIV morbidity and mortality, and in preventing new infections where the viral load among people living with HIV is suppressed [5]. Several barriers to the utilization of HIV services for people living with HIV pre-existed the advent of COVID-19 [7] and these may have been potentially intensified, with new barriers arising due to COVID-19. There is thus a great need to see how the outbreak of COVID-19 intensified or redefined health care utilization of HIV care and treatment services which are lifelong and are essential needs for healthy living of people living with HIV. There is a need to have studies that check if the gains toward reaching epidemic control of HIV were not or are still being threatened by the outbreak of COVID-19.

Globally, 39 million people were living with HIV and 27.5 million people were accessing ART in 2021 [8]. Of these, sub-Saharan Africa was home to two thirds (67%) of people living with HIV [9, 10]; Zimbabwe had a generalized HIV epidemic and was home to 1.3 million people living with HIV [9, 10]. Zimbabwe is still to ramp up its efforts to reach HIV epidemic control with a significant population of people on ART not utilizing all HIV care and treatment services such as viral load monitoring which has coverage of less of than 50% nationally [11]. In a study to ascertain the barriers to HIV service utilization by people living with HIV in two provinces of Zimbabwe, it was found that client-related barriers, community-related barriers, and health system barriers were critical in impeding utilization [7]. In another study that sought to understand the predictors of retention of HIV in Zimbabwe, it was found that being an adolescent or a young adult, receiving care at a primary health care facility, having initiated ART between 2014 and 2015, and having World Health Organization Stage IV, predicted attrition in HIV care [12].

Since the outbreak of COVID-19, Zimbabwe has recorded 264,127 confirmed COVID-19 cases and 5,668 deaths in the second week of March 2023 [13]. A national lockdown was introduced on 30 March 2020 [8, 14]. Another nationwide lockdown was initiated after the festive season in January 2021. Despite limitation on human movements, the lockdown restrictions resulted in stoppages of non-emergency medical care at health institutions. More so, there were disruptions in supply chains of consumables for medical care [15]. This study examined the impact of the COVID-19 pandemic on the utilization of HIV care and treatment services among people living with HIV who were on ART and the factors associated with it in Zimbabwe.

## Methods and Data

### Data Source and Study Design

The study utilized a concurrent triangulation design of which only one data collection phase was used through three different modalities. The quantitative data was collected from people living with HIV on ART through a structured interviewer-administered questionnaire. The quantitative questionnaire asked about all the predisposing, enabling, need, and environmental factors as they related to accessing or not accessing the expected HIV care and treatment services per each sample person living with HIV on ART. The questionnaire was administered to adolescents and young people (15–24) by Community Adolescent Treatment Supporters from Zvandiri, while data for adults (25 +) was administered by Community Health Agents from Zimbabwe National Network of People Living with HIV (ZNNP +). A total of 2,157 people living with HIV were sampled for the study of which 791 were adolescents and young people (15–24) and 1,366 were adults (25 +). On the qualitative data collection modality, qualitative data was collected through in-depth interviews with purposively sampled key stakeholders such as 24 HIV volunteers, six district HIV focal persons and six health promotion officers. In the qualitative phase, in-depth and key informant interviews were done using a semi-structured interview guide. The semi-structured guide asked for information related to the challenges of accessing HIV services and COVID-19 vaccinations during the COVID-19 period among people living with HIV. This information provided more understanding about the factors. The analysis of the quantitative and the qualitative data were conducted separately but concurrently.

## Study Variables

### Outcome Variable

The dependent variable for this study was utilization of HIV care and treatment services. The outcome variable was defined as utilization of HIV care and treatment services which consisted of four services, namely, ART drug refill, viral load monitoring, TB preventative therapy and TB screening. Each of the outcome variables was coded as binary with “1” representing utilization of HIV care and treatment services and “0” representing non-utilization of HIV care and treatment services during the COVID-19 period.

### Independent Variables

**Fig. 1:**
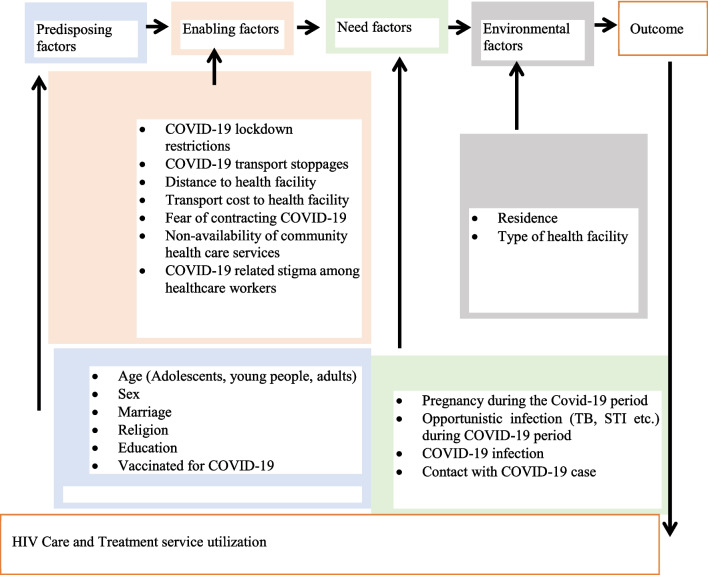
The health utilization model for HIV Care and treatment services in the context of COVID-19 in Zimbabwe

The study was premised on the fifth version of the Anderson health care services utilization model. This model argues that the actual use of health care services is a function of three factors classified as predisposing, enabling, and need factors. Firstly, the predisposing factors include socio-cultural characteristics of individuals that exist prior to their illness. These include demographic characteristics such as age and gender and social structural variables such as education, among others. Secondly, the enabling factors are those that involve the logistical aspects of obtaining care such as the resources available, whether individually or in a community. These may include the individual or family applying the means and know how to access health services, income, health insurance, a regular source of care, travel, and extent and quality of social relationships. At the community level, these may imply available health personnel and facilities and waiting time, among others. Thirdly, the need factors are the most immediate causes of health service use, including functional and health problems that generate the need for health care services. Figure 1 shows how the Anderson Model was adopted to explain the predictors of HIV services utilization in the context of COVID-19.

The predisposing factors included: age (15–19, 20–24, 25–39, 40–54, 55 +), sex (male, female) marriage (not married, married), religion (Roman Catholic, Apostolic Sect, Pentecostal/Protestant, other religions), education (primary, secondary, tertiary, do not know/did not attend school/no answer), COVID-19 vaccination (yes/no). The enabling factors included: COVID-19 lockdown restrictions, COVID-19 transport stoppages, distance to health facility, transport cost to health facility, fear of contracting COVID-19, non-availability of community health care services, and COVID-19 related stigma among health care workers. The need factors included: opportunistic infection during COVID-19 period (yes/no), COVID-19 infection (yes/no), and contact with COVID-19 case (yes/no) while environmental factors included: residence (urban /rural) and type of health facility (central /provincial health facility, district health facility, primary health care facility).

### Statistical Analysis

Statistical analysis was performed using Stata version 14 software with 5% level of significance. Frequency distributions and the Chi-square test of association were used at the univariate and bivariate levels of analysis. Multivariate binary logistic regression was used to identify the predictors of utilizing HIV care and treatment services. All quantitative analyses were conducted at α = 0.05. Adjusted odds ratios (aOR) with corresponding p-values were reported. All covariates from the bivariate analysis were included in the multivariate analysis regardless of their significance. Qualitative analysis was undertaken using the NVIVO software (version 12). The NVIVO software is a software that allows for the collaboration and organisation of text data. Therefore, to review more significant effects from the in-depth interviews conducted in this study, NVIVO was used. As we conducted the interviews, we jotted down notes from the responses to the interview guide. We then created summaries and assigned codes to the data with the aid of the NVIVO software. We then categorised the codes and sorted the main themes that related to the challenges of accessing HIV services and COVID-19 vaccination and the reasons for not accessing HIV services by predisposing, enabling, need, and environmental factors.

### Ethical Considerations

Ethics approval was granted by the Medical Research Council of Zimbabwe under reference: *MRCZ/A/2868* and also the University of the Witwatersrand Ethics Committee (Medical) under reference number: *M220425.* Survey participants were guaranteed assurance of anonymity and confidentiality of their results. Furthermore, participation in the data collection process was voluntary.

## Results

### Socio-demographic characterIstics of Study Participants

A total of 2,157 people living with HIV aged 15 years and older who were on ART before the advent of COVID-19 were included in the study as reported in Table 1. The majority of the participants were adults aged 25 years and above (63.3%), while adolescents and young people were 36.7%. The majority of the participants were female (62.6%) while 37.4% were male. Nine hundred and seventy-eight (45.3%) were single, 42.2% were married and 12.4% were widowed. More than half of the study participants (63.7%) were from rural areas and 36.3% were from urban areas. Pentecostal/Protestant contributed to the majority representation with 34.96%. The majority (66.1%) had gone through secondary education. Most of the participants (69.9%) had access to their HIV services from primary health care facilities. The majority of the participants had been vaccinated for COVID-19 (85.8%).

**Table 1:** Distribution of socio-demographic characteristics of study participants (N = 2157)

Categories	Frequency (N)	Percentage (%)
Age		
15–19	326	15.11
20–24	465	21.56
25–39	513	23.78
40–54	604	28.00
55 +	249	11.54
Sex		
Male	806	37.37
Female	1351	62.63
Marital Status		
Not Married	1246	57.77
Married	911	42.23
Residence		
Rural	1374	63.70
Urban	783	36.30
Religion		
Roman Catholic	225	10.43
Apostolic sect	475	22.02
Pentecostal/Protestant	754	34.96
Other religions	703	32.96
Education		
Primary	492	22.81
Secondary	1426	66.11
Tertiary	129	5.98
Do not know/Did not attend school/No answer	110	5.10
Type of health facility where HIV services are being accessed		
Central/Provincial health facility	94	4.30
District health facility	556	25.70
Primary health care facility	1507	69.87
Vaccinated for COVID-19		
Yes	1851	85.81
No	306	14.19

### Utilization of HIV Services Among People living with HIV at the Advent of COVID-19

Table 2 shows the bivariate analysis of the proportion of utilization of HIV services among people living with HIV during COVID-19 restriction periods by predisposing, need, and environmental factors. The TB screening services had relatively lower utilization at 83% and above, and below 90% for TB preventative therapy across all predisposing, need, and environmental factors.

**Table 2:** Proportion of utilization of HIV services among people living with HIV during COVID-19 restrictions periods by predisposing, need and environmental factors (N = 2157)

Categories	Total	Number utilizing HIV services (N) and percent (%)			
		ART refill (%)	Viral load monitoring (%)	TB preventative therapy (%)	TB screening (%)
Predisposing factors					
Age		***	***	***	***
15–19	326	307 (94)	297 (91)	290 (89)	266 (82)
20–24	465	420 (90)	418 (90)	419 (90)	387 (83)
25–39	513	464 (90)	435 (84)	408 (80)	360 (70)
40–54	604	579 (96)	556 (92)	510 (84)	418 (69)
55 +	249	240 (96)	233 (94)	216 (87)	181 (72)
Sex					
Male	806	744 (92)	721 (89)	691 (86)	620 (77)
Female	1351	1266 (94)	1281 (90)	1152 (85)	992 (73)
Marriage					
Not married	1246	1152 (92)	1117 (90)	1059 (85)	931 (75)
Married	911	858 (94)	822 (90)	784 (86)	681 (75)
Religion		*	**	*	**
Roman Catholic	225	206 (92)	192 (85)	187 (83)	173 (77)
Apostolic sect	475	451 (95)	432 (91)	404 (85)	340 (72)
Pentecostal/Protestant	754	690 (92)	662 (88)	629 (83)	539 (71)
Other religions	703	663 (94)	653 (93)	623 (89)	560 (80)
Education		*	**		**
Primary	492	470 (96)	458(93)	426 (87)	349 (71)
Secondary	1426	1318 (92)	1265 (89)	1203 (84)	1065 (75)
Tertiary	129	116 (90)	112 (87)	114 (88)	102 (79)
Do not know/Did not attend school/No answer	110	106 (96)	104 (95)	100 (91)	96 (87)
Vaccinated for COVID-19		**	***	*	**
Yes	1851	1737 (94)	1683 (91)	1594 (86)	1405 (76)
No	306	273 (89)	256 (90)	249 (81)	207 (68)
Need factors					
Pregnancy in the past 24 months					
Yes	196	186 (94)	177 (90)	169 (86)	151 (77)
No	1155	1080 (93)	1041 (90)	983 (85)	841 (73)
Opportunistic infection in the past 24 months			**		***
Yes	247	235 (95)	234 (95)	220 (89)	125 (87)
No	1910	1775 (93)	1705 (89)	1623 (85)	13.97 (75)
COVID-19 infection in the past 24 months					
Yes	315	289 (92)	283 (90)	273 (87)	249 (79)
No	1842	1721 (93)	1656 (90)	1570 (85)	1363 (74)
Contact of a COVID-19 case in the past 24 months					
Yes	417	389 (93)	377 (90)	356 (85)	327 (78)
No	1740	1621 (93)	1562(90)	1487(85)	1285 (73)
Environmental Factors					
Residence		***	***	**	Ns
Rural	1374	1309 (95)	1259 (92)	1199 (87)	1032 (75)
Urban	783	701 (90)	680 (87)	644 (82)	580 (74)
Type of Health facility where HIV services are accessed		***	*		***
Central/Provincial health facility	94	82 (87)	83 (88)	82 (87)	53 (56)
District health facility	556	546 (98)	518 (93)	490 (88)	433 (78)
Primary health care facility	1507	1382 (92)	1338(89)	1271(84)	1126 (75)

***p < 0.001, **p < 0.01, *p < 0.05

### Predisposing Factors

Age was statistically significant at p = 0.00 at bivariate analysis across utilization of ART refill, viral load monitoring, TB preventative therapy and TB screening. We found that the age groups 20–24 and 25–39 contributed to the majority not utilizing (≥ 10%) for people living with HIV who had interruptions in accessing all the HIV services in this study which were accessing ART refill, viral load monitoring, TB preventative therapy, and TB screening. Older age groups from 40 years and older had higher utilization, such as ART refill and viral load monitoring with 96% utilization of ART refills and ≥ 92% on viral load monitoring. Religion was also statistically significant at the bivariate analysis with utilization of HIV services. In comparison to other religions and the apostolic sects, the study found a relatively lower utilization of ART refills in Roman Catholic (92%) and Pentecostal/Protestant (92%). The same trend was realized in TB preventative therapy with Roman Catholic (83%) and Pentecostal/Protestant (83%) utilization.

The study revealed that there was a lower proportion of utilization of all four HIV services among people living with HIV who were not vaccinated for COVID-19. The ART refills were 94% compared to 89% for the vaccinated and unvaccinated respectively. Education was also significantly associated with utilization of ART refills, viral load monitoring, and TB screening. The study revealed that there was a higher proportion of utilization of all four HIV services among people living with HIV who were vaccinated for COVID-19 than those not vaccinated. The ART refills were 94% compared to 89% for the vaccinated and unvaccinated respectively. This followed 91% and 90% for viral load monitoring, 86% and 81% for TB preventative therapy and 76% and 68% for TB screening. Being vaccinated for COVID-19 was significantly associated with utilization of HIV services.

### Need Factors

Females living with HIV who had been pregnant in the period of COVID-19 restrictions had higher utilization of HIV services. Regarding ART refills, pregnant females had a higher utilization of 94% compared to 93% for those that were never pregnant during the period. The same trend was similar for TB screening which was 77% and 73% for pregnant females and non-pregnant in the COVID-19 restriction period respectively. For viral load monitoring, the utilization proportion was similar at 90% for both, 86% and 85% respectively for TB preventive therapy. Opportunistic infections during the COVID-19 restriction period had relatively higher utilization proportion compared to people living with HIV who did not have opportunistic infections across all four HIV services. Opportunities infections were found statistically significant for utilization of viral load monitoring and TB preventative therapy. Thus, having an opportunistic infection and having no opportunistic infection respectively was 95% and 93% for ART refill, 95% and 89% for viral load monitoring, 89% and 85% for TB preventative therapy, and 87% and 75% for TB screening.

### Environmental Factors

Residence had significant association with utilization of ART refills, viral load monitoring, and TB preventive therapy services. We found higher proportions of utilization across all four HIV services in rural areas compared to urban areas in the COVID-19 restriction period. For ART refill, there was 95% utilization in rural areas and 90% in urban areas. In viral load monitoring, there was 92% utilization in rural areas and 87% in urban areas. In TB preventative therapy, there was 87% utilization in rural areas and 82% in urban areas. In TB screening, there was 75% utilization in rural areas and 74% in urban areas. Concerning the type of health facility, there was significant association with utilization of ART refills, viral load monitoring, and TB screening services. We found lower proportional utilization in central/provincial health facilities, then district health facilities and primary health facilities in that order. The TB screening utilization was 53% among people accessing services at central/provincial hospitals compared to 75% at primary health centers.

### Enabling Factors

**Fig. 2:**
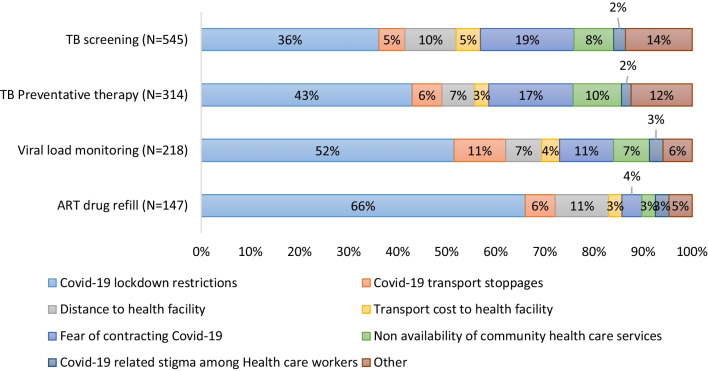
Enabling factors: Reasons for non-utilization of HIV services among people living with HIV during COVID-19 restriction periods

COVID-19 lock down restrictions were noted as the major reason for not utilizing all four HIV services as shown in Fig. 2. For ART refills, distance to the health facility contributed 11% of the reason for HIV non-utilization as it was the second majority reason after COVID-19 restrictions. For viral load monitoring, COVID-19 transport stoppages (11%) and fear of contracting COVID-19 (11%) were the second majority reasons after COVID-19 restrictions. For TB preventative therapy, fear of contracting COVID-19 (17%) and non-availability of community health workers in COVID-19 restrictions (7%) were the second and third majority reasons after COVID-19 restrictions respectively. For TB screening, fear of contracting COVID-19 (19%) was the second majority reason after COVID-19 restrictions.

From the in-depth interviews with key stakeholders, it was apparent that some HIV services that used to be provided in the community were halted. This was most apparent in periods of high COVID-19 infections.

“Community services in which ART was delivered in households or community viral load monitoring were difficult to conduct in COVID-19 times. This therefore meant some people living with HIV who used to enjoy these community services had to resort to coming to the facility where there was fear contracting COVID-19”. (Key stakeholder).

### Factors for Utilization of ART Refill Services During COVID-19 Restriction Periods

Our study findings revealed that adolescents aged 15–19 (aOR = 2.16; 95% CI: 1.18–3.96) had higher odds of utilizing ART refills compared to their counterparts who were aged 20–24 as reported in Table 3. Being a Pentecostal/Protestant (aOR = 0.53; 95% CI: 0.32–− 0.90) was associated with less likelihood of utilizing ART refills compared to the Apostolic Sect. Regarding environmental factors, being in a rural area was associated with higher odds of utilizing the ART refill service (aOR = 2.20; 95% CI: 1.49–3.24), than their urban counterparts. Sixty percent of the key informants were of the view that this was more likely as people in rural areas would have had less interruptions to accessing their routine HIV services as opposed to people in the urban areas.

“There was a smaller number of COVID-19 cases in rural areas compared to urban areas; COVID-19 was heavily urban hence the utilization challenges followed suit.” (Key informant interview)

**Table 3:** Multivariate logistic regression analysis of utilization of HIV services among people living with HIV during COVID-19 restrictions periods (N = 2157)

Categories	Adjusted odds ratio (AOR) and 95% confidence intervals (CI)			
	ART refill	Viral load monitoring	TB preventative therapy	TB screening
Predisposing factors				
Age				
15–19	2.16 (1.18–3.96) *	1.20 (0.72–2.04)	0.97 (0.59–1.58)	1.14 (0.76–1.72)
20–24	Ref			
25–39	0.69 (0.40–1.18)	0.41 (0.26–0.66)***	0.30 (0.20–0.47)***	0.32 (0.22–0.46)***
40–54	1.73 (0.97–3.12)	0.88 (0.54–1.43)	0.44 (0.29–0.68)***	0.30 (0.21–0.42)***
55 +	1.72 (0.74–3.93)	0.94 (0.49–1.84)	0.50 (0.29–0.86)*	0.34 (0.22–0.53)***
Sex				
Male	0.82 (0.56–1.20)	0.81 (0.59–1.11)	0.83 (0.63–1.09)	0.95 (0.76–1.20)
Female	Ref			
Marital Status				
Not married	0.73 (0.48–1.11)	0.81 (0.58–1.12)	0.64 (0.49–0.86)**	0.74 (0.59–0.93)*
Married	Ref			
Religion				
Roman Catholic	0.65 (0.33–1.26)	0.58 (0.34–0.97)*	0.87 (0.55–1.39)	1.23 (0.84–1.83)
Apostolic sect	Ref			
Pentecostal/Protestant	0.53 (0.32–0.90)*	0.69 (0.46–1.05)	0.87 (0.62–1.23)	0.95 (0.72–1.25)
Other religions	0.99 (0.56–1.72)	1.29 (0.83–2.03)	1.30 (0.90–1.87)	1.36 (1.01–1.82)*
Education				
Primary	1.02 (0.60–1.74)	1.27 (0.82–1.96)	1.12 (0.80–1.56)	0.95 (0.73–1.24)
Secondary	Ref			
Tertiary	1.13 (0.56–2.26)	0.92 (0.51–1.67)	1.35 (0.74–2.46)	1.31 (0.80–2.13)
Do not know/Did not attend school/No answer	1.41 (0.48–4.11)	1.41 (0.59–3.40)	1.23 (0.61–2.48)	1.79 (0.97–3.29)
Vaccinated for COVID-19				
Yes	1.55 (0.99–2.44)	1.97 (1.36–2.87)***	1.59 (1.12–2.25)**	1.89 (1.41–2.54)***
No	Ref			
Need factors				
Opportunistic infection in the past 24 months				
Yes	1.43 (0.71–2.89)	2.53 (1.31- 4.88)**	1.52 (0.94–2.47)	2.77 (1.80–4.26)***
No	Ref			
COVID-19 infection in the past 24 months				
Yes	0.72 (0.39–1.30)	0.86 (0.51–1.43)	1.13 (0.73–1.78)	1.01 (0.70–1.46)
No	Ref			
Contact of a COVID-19 case in the past 24 months				
Yes	0.94 (0.53–1.67)	0.87 (0.55–1.38)	0.76 (0.52–1.12)	1.04 (0.76–1.43)
No	Ref			
Environmental factors				
Residence				
Rural	2.20 (1.49–3.24)***	1.50 (1.09–2.08)*	1.58 (1.19–2.08)**	1.04 (0.82–1.32)
Urban	Ref			
Type of health facility where HIV services are being accessed				
District health facility	5.48 (2.07–14.50)**	1.50 (0.67–3.32)	1.18 (0.56–2.47)	3.94 (2.28–6.79)***
Central/Provincial health facility	Ref			
Primary health care facility	0.85 (0.40–1.81)	0.76 (0.37–1.58)	0.64 (0.32–1.28)	3.00 (1.82–4.94)***
Enabling factors				
COVID-19 lockdown restrictions	0.99 (0.51–1.94)	0.83 (0.43–1.55)	1.32 (0.76–2.29)	1.29 (0.81–2.43)
COVID-19 transport stoppages	Ref			
Distance to health facility	1.89 (0.74–4.82)	1.28 (0.58–2.86)	1.18 (0.62–2.23)	0.89 (0.51–1.91)
Transport cost to health facility	3.41 (0.89–13.10)	1.61 (0.53–4.93)	0.92 (0.41–2.09)	0.81 (0.63–3.06)
Fear of contracting COVID-19	4.54 (1.94–10.64)***	2.46 (1.21–5.00)**	2.38 (1.32–4.31)**	2.25 (1.39–3.66)**
Non-availability of community health care services	0.35 (0.12–1.05)	0.43 (0.17–1.10)	0.33 (0.16–0.69)**	0.99 (0.49–2.00)
COVID-19 related stigma among Health care workers	1.37 (0.32–5.91)	0.68 (0.19–2.41)	1.51 (0.51–4.40)	1.30 (0.58–2.93)
Other	1.86 (0.71–4.91)	1.01 (0.46- 2.22)	1.80 (0.93–3.47)	1.33 (0.78–2.29)

*Ref* reference category, *CI* confidence interval, *AOR* adjusted odds ratio

***p < 0.001, **p < 0.01, *p < 0.05

In-depth interviews gave reasons such as overcrowding in urban areas as the major drivers of HIV utilization challenges after the advent of COVID-19. Accessing health services at district health facilities was significantly associated with higher odds of accessing ART refills (aOR = 5.48; 95% CI: 2.07–14.50), than those accessing at central and provincial hospitals. Regarding enabling factors, the fear of contracting COVID-19 was significantly associated with accessing ART refills (aOR = 4.54; 95% CI 1.94–10.64). In the qualitative in-depth interviews, 70% of community volunteers felt that there was fear among people living with HIV that COVID-19 infection may have more severe outcomes.

“People living with HIV did not want anything that will lead to compromising their immune system.” (Community volunteer in-depth interview)

Hence, there was a push was to ensure that clients’ HIV services were always met, and they remained immune strong against COVID-19 if affected. All in-depth and key informant interviews did not find religion as having had an influence on access to HIV services during the COVID-19 period. Also, the community volunteers did not find sex a factor that influenced utilization of HIV services among people living with HIV after the advent of COVID-19.

### Factors for Utilization of Viral Load Monitoring Services During COVID-19 Restriction Periods

Our findings revealed that adults aged 25–39 (aOR = 0.41; 95% CI: 0.26–0.66) were less likely to utilize viral load monitoring compared to young people aged 20–24 as reported in Table 3. Being a Roman Catholic (aOR = 0.58; 95% CI: 0.34–0.97) was associated with less likelihood of utilizing viral load monitoring compared to the Apostolic Sect. Being vaccinated for COVID-19 was significantly associated with higher odds of utilizing viral load monitoring service (aOR = 1.97; 95% CI: 1.36–2.87) than those not yet vaccinated. Regarding need factors, having an opportunistic infection in the COVID-19 restriction period was significantly associated with higher odds of accessing viral load monitoring (aOR = 2.53; 95% CI: 1.31–4.88) than those who did not have an opportunistic infection. Regarding environmental factors, being in a rural area was associated with higher odds of utilizing viral load monitoring (aOR = 1.50; 95% CI: 1.09–2.08) than their urban counterparts. Regarding enabling factors, the fear of contracting COVID-19 was significantly associated with accessing viral load monitoring (aOR = 2.46; 95% CI 1.21–5.00), compared to COVID-19 transport stoppages.

### Factors for Utilization of TB Preventative Therapy Services During COVID-19 Restriction Periods

Our study findings revealed that, adults aged 25–39 (aOR = 0.30; 95% CI: 0.20–0.47) were less likely to utilize TB preventative therapy compared to young people aged 20–24 as reported in Table 3. Moreover, the adults aged 40–54 (aOR = 0.44; 95% CI: 0.29–0.68) were also less likely to utilize TB preventative therapy compared to young people aged 20–24. Similarly, adults aged 55 + (aOR = 0.50; 95% CI: 0.29–0.86) were also less likely to utilize TB preventative therapy compared to young people aged 20–24. Being unmarried (aOR = 0.64; 95% CI: 0.49–0.86) was associated with less probability of utilizing TB preventative therapy compared to the married counterparts. Being vaccinated for COVID-19 was significantly associated with higher odds of utilizing TB preventative therapy (aOR = 1.59; 95% CI: 1.12–2.25). Regarding environmental factors, being in a rural area was associated with higher odds of utilizing TB preventive therapy (aOR = 1.58; 95% CI: 1.19–2.08) than their urban counterparts. Regarding enabling factors, the fear of contracting COVID-19 was significantly associated with accessing TB preventative therapy (aOR = 2.38; 95% CI 1.32–4.31), compared to COVID-19 transport stoppages. Non availability of community health care services during the COVID-19 restriction period was significantly associated with less likelihood of accessing TB preventative therapy (aOR = 0.33; 95% CI 0.16–0.69).

### Factors for Utilization of TB Screening Services During COVID-19 Restriction Periods

Our findings revealed that adults aged 25–39 (aOR = 0.32; 95% CI: 0.22–0.46) were less likely to utilize TB screening compared to young people aged 20–24 as reported in Table 3. Moreover, adults aged 40–54 (aOR = 0.30; 95% CI: 0.21–0.42) were less likely to utilize TB screening compared to young people aged 20–24. Similarly, adults aged 55 + (aOR = 0.34; 95% CI: 0.22–0.53) were less likely to utilize TB screening compared to young people aged 20–24. People living with HIV who were not married (aOR = 0.74; 95% CI: 0.59–0.93) were less likely of utilizing TB screening compared to the married counterparts. Being a Pentecostal/Protestant (aOR = 0.53; 95% CI: 0.32–0.90) was associated with less likelihood of utilizing ART refills compared to the Apostolic Sect. Similarly, other religions (aOR = 1.36; 95% CI: 1.01–1.82) were associated with utilizing TB screening compared to the Apostolic Sect. Being vaccinated \for COVID-19 was significantly associated with higher odds of utilizing utilization of TB screening services (aOR = 1.89; 95% CI: 1.41–2.54) than those not yet vaccinated. Regarding need factors, having an opportunistic infection in the COVID-19 restriction period was significantly associated with higher odds of accessing TB screening (aOR = 2.77; 95% CI: 1.80–4.26) than those who did not have an opportunistic infection. Accessing health services at district health facilities was significantly associated with higher odds of accessing TB screening (aOR = 3.94; 95% CI: 2.28–6.79) than those accessing at central and provincial hospitals. Accessing health services at primary health facilities was significantly associated with higher odds of accessing TB screening (aOR = 3.00; 95% CI: 1.82—4.94) than those accessing at central and provincial hospitals. Regarding enabling factors, the fear of contracting COVID-19 was significantly associated with accessing TB screening (aOR = 2.25; 95% CI 1.39–3.66) compared to COVID-19 transport stoppages.

In the qualitative findings, 50% of community volunteers felt that opportunistic infections such as TB that happened during the COVID-19 pandemic period were also an important factor in pushing less people living with HIV to access HIV services as their health facilities were nursing COVID-19 patients.

*At the peak of the COVID-19 pandemic it was not easy to make it to your appointment, at your hospital that you know many COVID-19 patients are being taken care of. In most cases if you had an opportunistic infection, you will be forced to take on the risk. (Community volunteer in-depth interview)*

Hence with in this instance opportunistic infection became a push factor to ensure HIV services are utilised as noted by the community volunteers.

## Discussion

The study was conducted to explore the predictors of continued utilization of HIV care and treatment services since the COVID-19 outbreak among people living with HIV on ART in Zimbabwe. Regarding the COVID-19 outbreak effects on utilization of HIV care and treatment services, the study found that 7% people living with HIV who were part of the study had interruptions in accessing their ART refills. In terms of accessing routine viral load monitoring, 10% of the people living with HIV reported an interruption. Moreover, 14% of the people living with HIV eligible for TB preventative therapy also reported an interruption in accessing the service. Routine TB screening had the highest proportion of interruptions at 25%. COVID-19 lockdown restrictions were noted as the main reason for not accessing services as expected during the COVID-19 curtailing measures. Concerning the predictors for utilization of HIV services at the advent of COVID-19, in multivariate analysis, some categories of age, marital status, religion, COVID-19 vaccination, opportunistic infections, residence, type of facility, and fear of contracting COVID-19 had a significant likelihood for HIV services utilization at the advent of COVID-19.Our study found that study participants could not meet their clinical appointments to get ART refills at the advent of COVID-19 due to COVID-19 restrictions. The finding that COVID-19 was a barrier to visiting health facilities for ART refills was consistent with what was found in studies conducted in different countries and regions [16–20]. However, in a study in South Africa, the influence of COVID-19 on accessing ART refills was not found [21]. This study also found an increased proportion of non-utilization of TB services, such as accessing TB preventative therapy and TB screening among people living with HIV when compared to ART refills and viral load. This was also consistent with several studies that noted a decline in the utilization of TB services [22, 23]. This study therefore confirmed this for the people living with HIV who require the TB services to guard against HIV and TB comorbidity. The reasons for low utilisation of TB services among people living with HIV was explained in the qualitative in-depth interviews in two ways. Firstly, among the key informants this was noted as a function of being in an emergency mode, whereby the people living with HIV had to prioritise the services that they deemed most important. The ART drug refills became the most important and they would not prioritise getting TB screening or their TB preventative therapy course. Secondly, among the community volunteer in-depth interviews, TB symptoms were similar to COVID-19 symptoms and this was explained as a barrier. Hence, there was some level of stigma towards accepting services such as TB screening.

Our findings revealed that residence was an important factor in defining utilization of HIV services at the advent of COVID-19. This result was also similar to a study in Rwanda, which found residence significant in the access to ART refills [24]. Our findings did not find enabling factors such as distance to health facility as significant in increasing the odds of HIV services utilization at the advent of COVID-19. This was also consistent with a study in Uganda, which found distance statistically insignificant but the WHO staging and obesity as significant [25]. Our study also found residence a significant factor in utilization of HIV services during the COVID-19 period. This could be because COVID-19 cases were more prevalent in urban areas. Therefore, COVID-19 restrictions affected people living with HIV more in urban areas than rural areas. As a predisposing factor, being vaccinated for COVID-19 was significantly associated with a high likelihood of utilization of viral load monitoring, TB preventative therapy, and TB screening. It therefore follows that those people who had taken the COVID-19 vaccination would routinely access their HIV services as well. This could be attributed to health seeking behaviours. Chances are that people living with HIV who utilized the opportunity to get COVID-19 vaccinated quicker, are also the ones that routinely access their HIV services, such as viral load monitoring, TB preventative therapy, and TB screening. This utilization of HIV services study makes two unique contributions to the research literature. First, previous studies on difficulties in accessing HIV services and sexual reproductive services during the COVID-19 pandemic have largely focused on documenting the effects of COVID-19 without exploring the predictors that defined utilization of the services at the advent of COVID-19 [17, 24, 26–30]. This study provides understanding about how predisposing, need, environmental, and enabling factors are associated with defining the utilization of HIV services during the COVID-19 period. Secondly, the study provided reasons from the people living with HIV on what factors were barriers for them in accessing HIV services during the advent of COVID-19. Most importantly, the results of the multivariate regression analysis showed that there were higher odds of accessing HIV services at the advent of COVID-19 among older age bands compared to adolescents aged 15–19. This finding raises a significant public health concern about HIV; to achieve HIV epidemic control, there is a need to prioritize interventions for adolescents to always keep utilization of health services in this age group in check. Although the severity of the COVID-19 pandemic has dwindled, it appears to come and go in waves, and some few countries are still having relatively high cases. It is therefore likely that the factors associated with utilization of HIV services identified by the study, such as age, residence, type of health facility, and fear of contracting COVID-19, among others need to be included when planning improved access to health utilization. In the event of any disease outbreak, these factors may need to be prioritized in crafting informed public health responses. In terms of limitations, it is important to note that participants in this study do not reflect the entire population of people living with HIV. The study’s main strength was that it used a relatively large representative of more than 2,000 people living with HIV.

## Conclusion

The study has established that COVID-19 restrictions impacted utilization of HIV care and treatment services among people living with HIV in Zimbabwe. We found that age, marital status, religion, education, being vaccinated for COVID-19, opportunistic infections, residence, type of health facility, fear of contracting COVID-19, and non-availability of community health services were significantly associated with utilization of one or more HIV services. There is now more than ever a greater focus on the global effort to end the HIV/AIDS epidemic through achieving epidemic control. There is thus a need to ensure that there is access to and uptake of HIV treatment services for all populations across ages, genders, religions, and localities, including those who are vulnerable despite the challenges proffered by new disease emergencies. The Zimbabwe Ministry of Health and Child Care has affirmed its commitment to epidemic control in the country. It is thus imperative that the implications of COVID-19 on HIV care and treatment that were investigated provide useful information on utilization predictors in times of intersecting epidemics and that the HIV response also needs tightening in the last mile of achieving epidemic control. Future studies may need to integrate how emerging predictors such as mental health among people living with HIV weigh in on the utilization of HIV services, particularly in emergencies such as COVID-19.
